# Effects of exposure to 5-MeO-DIPT during adolescence on brain neurotransmission and neurotoxicity in adult rats

**DOI:** 10.1007/s11419-018-0433-x

**Published:** 2018-07-19

**Authors:** Karolina Noworyta-Sokołowska, Katarzyna Kamińska, Joanna Rzemieniec, Agnieszka Wnuk, Jakub Wojcieszak, Anna Maria Górska, Grzegorz Kreiner, Małgorzata Kajta, Krystyna Gołembiowska

**Affiliations:** 1 Department of Pharmacology, Institute of Pharmacology, Polish Academy of Sciences, 12 Smętna, 31-343 Kraków, Poland; 2 Department of Experimental Neuroendocrinology, Institute of Pharmacology, Polish Academy of Sciences, 12 Smętna, 31-343 Kraków, Poland; 3 Department of Pharmacodynamics, Medical University of Łódź, Muszyńskiego 1, 90-151 Łódź, Poland; 4 Department of Brain Biochemistry, Institute of Pharmacology, Polish Academy of Sciences, 12 Smętna, 31-343 Kraków, Poland

**Keywords:** 5-MeO-DIPT, Exposure in adolescence, Neurotransmitter release, Dopamine, serotonin and glutamate, DNA damage, Cytotoxicity

## Abstract

**Purpose:**

Tryptamine hallucinogen 5-methoxy-*N,N*-diisopropyltryptamine (5-MeO-DIPT) is a serotonin transporter inhibitor with high affinity for serotonin 5-HT_1A_ and 5-HT_2A/C_ receptors. We showed previously that 5-MeO-DIPT in a single dose increased neurotransmitter release in brain regions of rats and elicited single- and double-strand DNA breaks. Herein we investigated the effects of repeated-intermittent 5-MeO-DIPT administration in adolescence on dopamine (DA), serotonin (5-HT) and glutamate release in brain regions of adult rats. Furthermore, we examined caspase-3 activity, oxidative DNA damage, the *Gpx3, Sod1, Ht1a* and *Ht2a* mRNA expression levels, and cell viability.

**Methods:**

Neurotransmitter release was measured by microdialysis in freely moving animals. Caspase-3 activity was assessed colorimetrically, and oxidative DNA damage with the comet assay, while the *Gpx3, Sod1, Ht1a* and *Ht2a* mRNA expression levels were assessed by real-time polymerase chain reaction. Cell viability was studied in SH-SY5Y and Hep G2 cells by the MTT test.

**Results:**

We observed changed responses of DA, 5-HT and glutamate neurons to a challenge dose of 5-MeO-DIPT when animals were treated repeatedly in adolescence with this hallucinogen. The basal extracellular levels of DA and 5-HT were decreased in the striatum and nucleus accumbens, while glutamate level was increased in the nucleus accumbens and frontal cortex. The damage of cortical DNA, increased *Gpx3* and *Sod1* mRNA expression and affected caspase-3 activity were also observed. Furthermore, decreased *Ht1a* and *Ht2a* mRNA expression in the frontal cortex and marked cytotoxicity of 5-MeO-DIPT were found.

**Conclusions:**

These results suggest that 5-MeO-DIPT given repeatedly during adolescence affects brain neurotransmission and shows neurotoxic potential observed in adult animals.

## Introduction

5-Methoxy-*N,N*-diisopropyltryptamine (5-MeO-DIPT) synthesized by Shulgin and Carter [1] is a tryptamine hallucinogen with a street name “foxy” or “foxy methoxy”. As a substitute for MDMA, 5-MeO-DIPT has been permanently controlled in the USA as a schedule I substance under the Controlled Substances Act (69 FR 58050) [2, 3].

5-MeO-DIPT is a competitive serotonin transporter (SERT) inhibitor and has a lower affinity for dopamine transporter (DAT) [4]. The hallucinogenic activity of 5-MeO-DIPT shown in mice was caused by the stimulation of postsynaptic 5-HT_2A_ receptors [5]. Moreover, 5-MeO-DIPT had also in vitro high affinity for 5-HT_1A_ and 5-HT_2C_ receptors [5, 6]. High concentrations of 5-MeO-DIPT produced a marked cytotoxicity assessed by a cell viability assay in COS-7 cells [3]. Chronic 5-MeO-DIPT administration decreased 5-HT levels in the rat prefrontal cortex, striatum and hippocampus [7–9], which may suggest its neurotoxicity. Clinical data indicated potent multi-organ effects of 5-MeO-DIPT as the users experienced euphoria, disinhibition, increased sociability, and visual and auditory hallucinations, but also effects like mioclonus, restlessness, insomnia, anxiety, nausea, vomiting and diarrhea [10]. According to the Drug Enforcement Administration (DEA), the doses used by human subjects range from 6 to 20 mg with the duration of symptoms for approximately 6 h [2]. The threshold dose for hallucinogenic activity was 4 mg with an effective range from 6 to 10 mg [11]. Our earlier microdialysis study in rats showed that 5-MeO-DIPT in a wide range of doses (5, 10 and 20 mg/kg) increased extracellular levels of dopamine (DA), serotonin (5-HT) and glutamate with different potencies among brain regions [12]. The decrease in DA, 3,4-dihydroxyphenylacetic acid (DOPAC) and homovanilic acid (HVA) tissue contents as well as DNA damage also suggested the neurotoxicity of 5-MeO-DIPT [12]. The study also supported the idea that the hallucinogenic activity of 5-MeO-DIPT is mediated through 5-HT_1A_ and 5-HT_2A_ receptors, because 5-MeO-DIPT induced head-twitch response and potentiated forepaw treading induced by 7-(dipropylamino)-5,6,7,8-tetrahydronaphthalen-1-ol (8-OH-DPAT) [13].

Reports by the European Monitoring Centre for Drugs and Drug Addiction (EMCDDA) [14] have indicated an increased abuse of new psychoactive substances (NPS) by adolescents. Exposure to NPS during adolescence may have an impact on brain development and may lead to neuropsychiatric disorders in adulthood. Several studies in adolescent animals showed their greater addictive potential in behavioral studies with cocaine [15] or ethanol [16]. Adolescent rats were also more sensitive than adult animals for cataleptic effect of haloperidol [17, 18] or hyperlocomotion induced by morphine [19]. The ontogenetic variations in drug response may result from age-related differences in pharmacokinetics, drug metabolism and excretion rates [20]. It was shown that exposure to ethanol and cocaine during adolescence significantly increased aggressive behavior in adult rodents [16, 21]. Moreover, adults who used marijuana before 15 years of age were 6 times more likely to be dependent on an illicit drug than adults who first used marijuana at 21 years of age or older [22]. The age of first NPS use may be a critical factor for illicit drug use in adulthood. For instance, adults who used marijuana before 15 years of age reported lifetime cocaine and heroin use and nonmedical use of other drugs [22]. Thus, NPS exposure during adolescence may result in severe, long-term and adverse effects in adult life. Unfortunately, there are no direct follow-up human studies in the literature of subjects abusing NPS in adolescence and the effects in adulthood.

The aim of the present study was to find out whether exposure of rats to repeated doses of 5-MeO-DIPT during adolescence [postnatal days (PND) from 30 to 40] may have impact on brain neurotransmission in adulthood. The frontal cortex is the main target of hallucinogens, while the striatum and nucleus accumbens receive strong inputs from the frontal cortex [23]. Thus, 5-MeO-DIPT may stimulate neurons in the frontal cortex and, consequently, may affect striatal and accumbal neurotransmission, as shown by us previously [12]. Therefore, we measured the release of DA, 5-HT and glutamate in the rat striatum, nucleus accumbens and frontal cortex using microdialysis in freely moving adult animals pretreated with 5-MeO-DIPT during adolescence. The comet assay and caspase-3 activity test were performed to asses 5-MeO-DIPT genotoxic and proapoptotic properties, while the mRNA expression levels of *Gpx3* and *Sod1* genes were examined to get insight into brain defense system activation by 5-MeO-DIPT. The expression of 5-HT_1A_ and 5-HT_2A_ receptors was measured to determine long-term changes induced by 5-MeO-DIPT on serotonergic neurotransmission as these receptors are important for hallucinogenic effect [23]. Cell viability was also studied to assess 5-MeO-DIPT cytotoxicity.

## Materials and methods

### Animals

The study was carried out on male Wistar-Han rats (Charles Rivers, Sulzfeld, Germany) weighing 280–300 g. The animals arrived at the vivarium on the 21st day of age (PND) and were allowed to acclimate until PND 30 (9 days); then they were randomly assigned to control and drug-treated groups. The animals were housed in groups of 5 each in temperature (22 ± 1°C) and humidity (50–60%) controlled rooms under a 12 h light/12 h dark cycle (light phase beginning at 6 a.m.) and had free access to tap water and standard laboratory food (VRF 1; Special Diets Services, Witham, UK).

### Drugs and reagents

5-MeO-DIPT was purchased from Toronto Research Chemicals Inc. (Toronto, Canada). The chemicals used for high-performance liquid chromatography (HPLC) were obtained from Merck (Warsaw, Poland); ketamine hydrochloride and xylazine hydrochloride from Biowet (Puławy, Poland); the chemicals used for the comet assay were from Trevigen (Gaithersburg, MD, USA); Triton from SERVA Electrophoresis (Heidelberg, Germany); the RNeasy Mini Kit from Qiagen (Valencia, CA, USA); the high capacity cDNA-reverse transcription kit and TaqMan probes for specific gene encoding of *HT1a, HT2a, Gpx3, Sod1* and *β*-*actin* from Life Technologies Applied Biosynthesis (Foster City, CA, USA); probe qPCR Master Mix (2 x) from EURx (Gdańsk, Poland); cell culture media including the Dulbecco's modified Eagle's medium (DMEM) and DMEM/F12, heat inactivated fetal bovine serum (FBS), phosphate buffered saline (PBS), trypsin–EDTA, penicillin and streptomycin from Life Technologies (Warsaw, Poland); dimethyl sulfoxide (DMSO), [3-(4,5-dimethyl-2-thiazolyl)-2,5-diphenyl-2*H*-tetrazolium bromide] (MTT), *o*-phthalaldehyde (OPA) and (+)-methamphetamine from Sigma-Aldrich (Poznań, Poland). Other common chemicals used were of the highest purity commercially available.

### Treatment

**Fig. 1:**
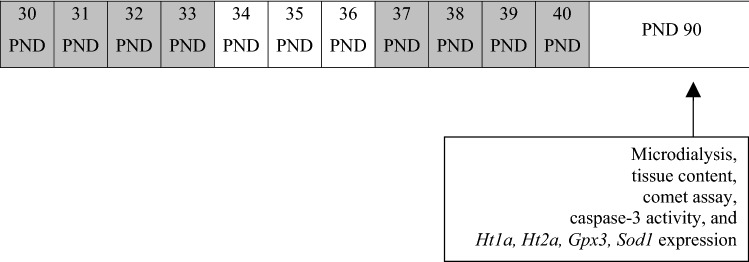
Schematic illustration of repeated-intermittent 5-methoxy-*N*,*N*-diisopropyltryptamine (5-MeO-DIPT; 8 × 2.5 mg/kg) administration during adolescence in rats. Grey areas indicate days of administration. *PND* postnatal day

Administration of 5-MeO-DIPT started when rats attained PND 30. Rats were injected with 5-MeO-DIPT once daily in a dose of 2.5 mg/kg for 4 days (PND 30–33) representing the early adolescence period, and after a 3-day break, another 4-day administration started (PND 37–40) representing the middle adolescence period [7]. That pattern of administration was based on drug use by adolescent humans, while the dose selection was based on our behavioural study (Fig. 1). All the experiments were performed when rats reached 90 PND. 5-MeO-DIPT was dissolved in a 0.9% NaCl and was administered subcutaneously (s.c.) to prevent abdominal irritation induced by chronic drug administration. The control groups received the corresponding volume of a 0.9% NaCl according to the same administration schedule as in the 5-MeO-DIPT-treated animals.

### Brain microdialysis

Animals were anesthetized with ketamine (75 mg/kg) and xylazine (10 mg/kg), and vertical microdialysis probes (AgnTho’s AB, CNS probes, MAB 4.15.4.Cu; MAB 4.15.3.Cu and MAB 4.15.2.Cu; AgnTho’s, Lidingö, Sweden) were implanted into the striatum, frontal cortex and nucleus accumbens, respectively, using the following coordinates: AP + 1.8, L − 3.0, V − 7.0; AP + 2.8, L − 0.8, V − 6.0; AP + 1.6, L − 1.1, V − 8.0; from the dura, respectively [24]. The number of animals for each treatment group and brain structure was four for the saline/saline group and six for the 5-MeO-DIPT treatment group. On the next day, probe inlets were connected to a syringe pump (BAS, West Lafayette, IN, USA), which delivered artificial cerebrospinal fluid composed of (mM) NaCl 147, KCl 2.7, MgCl_2_ 1.0 and CaCl_2_ 1.2; pH 7.4 at a flow rate of 2 μL/min. After 2 h of washout period, four basal dialysate samples were collected every 20 min; then animals were injected s.c. with 5-MeO-DIPT as indicated in the figure captions and fraction collection continued for 240 min. At the end of the experiment, the rats were sacrificed and their brains were histologically verified for the proper probe placement.

### Extracellular concentrations of DA, 5-HT and glutamate

The DA and 5-HT concentrations in dialysate fractions were analyzed by HPLC with electrochemical detection. Chromatography was performed using an Ultimate 3000 System (Dionex, Sunnyvale, CA, USA), electrochemical detector Coulochem III (model 5300; ESA, Chelmsford, MA, USA) with a 5020 guard cell, a 5014B microdialysis cell and a Hypersil Gold C18 analytical column (100 × 3 mm, particle size 3 μm; Thermo Fischer Scientific, Waltham, MA, USA). The mobile phase was composed of 0.1 M potassium phosphate buffer adjusted to pH 3.6, 0.5 mM Na_2_EDTA, 16 mg/L 1-octanesulfonic acid sodium salt and 2% methanol. The flow rate during analysis was set at 0.7 mL/min. The applied potential of a guard cell was 600 mV, while those of microdialysis cells were *E*_1_ = −50 mV, *E*_2_ = 300 mV with a sensitivity set at 50 nA/V. The chromatographic data were processed by the Chromeleon v. 6.80 (Dionex) software package run on a personal computer. The limit of detection of DA and 5-HT in dialysates was 0.002 pg/10 µL for DA and 0.01 pg/10 µL for 5-HT.

Glutamate levels in the extracellular fluids were measured electrochemically after derivatization with OPA/sulfite reagent to form isoindole-sulfonate derivative [25]. Chromatography was performed using an Ultimate 3000 pump (Dionex), LC-4B amperometric detector with a cross-flow detector cell (BAS) and an HR-80 column (80 × 4.6 mm, particle size 3 μm; ESA Inc, Chelmsford, MA, USA). The mobile phase consisted of 100 mM monosodium orthophosphate at pH 4.6 and 4% methanol. The flow rate was 1 mL/min and the applied potential of a 3-mm glassy carbon electrode was set at +600 mV at a sensitivity of 5 nA/V. Glutamate-derivative peak was compared with the respective standard and the data were processed using Chromax 2005 (Pol-Lab, Warszawa, Poland) software on a personal computer. The limit of detection of glutamate in dialysates was 0.03 ng/10 µL.

### Comet assay

#### Preparation of nuclear suspension

Animals were scarified by decapitation 60 (PND 90) days after termination of drug treatments. Each group (control and 5-MeO-DIPT-treated) comprised six animals. The frontal cortex was separated in anatomical borders. Next, the brain tissue was minced with a surgical scalpel and homogenized in a manual homogenizer with homogenizing solution containing 0.25% Triton. The homogenate was filtered and centrifuged at 850 × *g* for 10 min. Thereafter, the supernatant was discarded, while the pellet was resuspended in the same volume of homogenization medium without Triton and centrifuged for 10 min at 850 × *g*. The sediment was washed once more in the same way and centrifuged at 600 × *g* for 8 min. The pellet was resuspended in 0.8 mL of homogenization solution without Triton, mixed with 4.2 mL of purification medium and centrifuged at 19,000 × *g* for 45 min. The nuclei were obtained as a transparent sediment at the bottom. The pellet was resuspended in 0.5 mL of 2.0 M sucrose and was layered over a sucrose gradient (2.6, 2.4 M bottom to top). The gradient was allowed to stand for 3 h at 0°C before use. Fractionation of the nuclei was achieved by centrifugation at 19,000 × *g* for 45 min.

#### Analysis of DNA damage

The nuclei were added to a tube with 200 μL of PBS (without Ca^2+^ and Mg^2+^) and mixed gently. The suspension was mixed with low melting point agarose and transferred immediately onto comet slides. The slides were placed at 4°C in the dark for 10 min. Then the slides were immersed in pre-chilled lysis solution and left at 4°C in the dark for 30 min. The buffer was drained, the slides were immersed in alkaline unwinding solution and were left for 45 min in the dark. In the next step, electrophoresis was run at 21 V for 30 min. After electrophoresis, the slides were washed first with H_2_O, next with 70% ethanol and dried at 45°C for 10 min. The slides were then covered with dye and allowed to dry completely at room temperature in the dark. On the next day, the slides were examined under a fluorescent microscope. DNA damage was presented as the Olive tail moment, which is defined as the product of the tail length and the fraction of total DNA in the tail. Tail moment incorporates a measure of both the detectable size of migrating DNA (reflected in the comet tail length) and the number of damaged pieces (represented by the intensity of DNA staining in the tail). The Olive tail moment is calculated according to the formula: Olive tail moment = (tail.mean − head.mean) × tail %DNA/100.

### Assessment of caspase-3 activities

Animals were scarified by decapitation (*n* = 6, for control and 5-MeO-DIPT-treated groups) on PND 90. Brains were separated and brain regions were dissected along anatomical borders. The assessment of caspase-3 activity was performed as previously described by [26]. Tissues were homogenized in solution composed of radioimmunoprecipitation assay buffer and protease inhibitors for 20 min in 15,000 × *g*. The supernatant was incubated at 37°C using a colorimetric substrate preferentially cleaved by caspase-3, i.e., Ac-DEVD-pNA (*N*-acetyl-Asp-Glu-Val-Asp-*p*-nitro-anilide). The levels of *p*-nitroanilide were continuously monitored for 60 min using a Multimode Microplate Reader Infinite M200PRO (Tecan, Mannedorf, Switzerland). The data were analyzed using Magellan software, normalized to the absorbance of control animals and expressed as a percentage of control ± standard error of the mean (SEM) from three to four independent experiments. The absorbance of blanks, i.e., no-enzyme controls, was subtracted from each value.

### Real-time polymerase chain reaction

Animals were scarified by decapitation on PND 90 (*n* = 6, for control and 5-MeO-DIPT-treated groups). Brains were isolated and brain regions were dissected along anatomical borders. Total RNA was isolated from the frontal cortex and striatum using the RNeasy Mini Kit (Qiagen) according to the manufacturer’s protocol. Real-time polymerase chain reaction (PCR) procedure was adopted from [27]. The quantity of RNA was spectrophotometrically determined at 260 nm and 260/280 nm (ND/1000 UV/Vis; Tecan NanoDrop, San Jose, CA, USA). Two-step real-time PCR was performed. Both reverse transcription (RT) and the quantitative PCR (qPCR) assays were performed using the CFX 96 Real-Time PCR Detection System (Bio-Rad, Hercules, CA, USA). The RT reaction was performed at a final volume of 20 μL using 300 ng of RNA (as a cDNA template) and the High Capacity cDNA Reverse Transcription Kit (Applied Biosystems, Waltham, MA, USA), according to the manufacturer’s instructions. The products of the RT reaction were amplified using the TaqMan Gene Expression Master Mix containing TaqMan probes (TaqMan, Waltham, MA, USA) as primers specific to the genes encoding *HT1a, HT2a, Gpx3, Sod1* and *β*-*actin*. Amplification was performed in a total volume of 20 μL of the mixture containing 10 μL of the TaqMan Gene Expression Master Mix and 1.0 μL of the RT product as the PCR template. The reaction used TaqMan forward and reverse primers and probes labeled with the fluorescent reporter dye fluorescin amidite at the 5′-end and a quenching dye at the 3′-end. The standard qPCR procedures were performed: 2 min at 50 °C and 10 min at 95 °C, followed by 40 cycles of 15 s at 95 °C and 1 min at 60 °C. The threshold value (*C*_t_) for each sample was set during the exponential phase, and the delta delta *C*_t_ method was used for data analysis. The gene encoding *β*-*actin* was used as a reference gene.

### Cell cultures

SH-SY5Y cells purchased from the Leibniz Institute DSMZ-German Collection of Microorganisms and Cell Cultures (DSMZ, Braunschweig, Germany) were cultured in DMEM/F12 medium supplemented with 10% FBS and penicillin (100 U/mL)-streptomycin (100 μg/mL) at 37 °C in a humidified atmosphere enriched with 5% CO_2_. Upon reaching 80–90% confluency, cells were harvested with 0.25% trypsin in 1 mM EDTA and transferred into 96-well microplates at the density of 10,000 cells/well. Hep G2 cells were also obtained from DSMZ and cultured in DMEM medium supplemented with 10% FBS and penicillin (100 U/mL)-streptomycin (100 μg/mL) at 37 °C in a humidified atmosphere enriched with 5% CO_2_. Upon reaching 80–90% confluency, cells were harvested with 0.25% trypsin in 1 mM EDTA and transferred into 96-well microplates at the density of 5000 cells/well.

### Cell viability

Cell viability and mitochondrial function were assessed through MTT reduction by mitochondrial dehydrogenases. Following overnight incubation, complete culture medium was removed and replaced by fresh medium without FBS and working solutions of the tested compounds, 5-MeO-DIPT or methamphetamine, were added into microplate wells. After 24-h exposure, a solution of MTT (0.5 mg/mL) was added and cells were incubated for an additional 3 h at 37 °C. After aspiration of culture medium, formazan crystals were dissolved in DMSO and absorbance, proportional to the number of cells with intact mitochondria, was measured at 570 nm using a Bio-Rad microplate reader model 680 (Bio-Rad).

### Data analysis

Repeated measures ANOVA followed by Tukey’s post hoc test was performed to analyze drug effect on DA, 5-HT and glutamate release in the rat brain regions. All obtained data were presented as a percent of each basal level assumed to be 100%. DNA damage in the comet assay was tested using one-way ANOVA followed by Tukey’s multiple comparison test. One-way ANOVA preceded by the Levene’s test of homogeneity of variances was used to determine overall significance in caspase-3 and qPCR experiments. Differences between saline and 5-MeO-DIPT groups were assessed using a post hoc Newman-Keuls test. Cell viability measurements were analyzed using one-way ANOVA followed by Dunnett’s post hoc test. The median effective concentration (EC_50_) values were determined using non-linear regression from the plot of % viability against log dose of complexes added, using GraphPad Prism 6.0 software (GraphPad, San Diego, CA, USA). Differences were considered statistically significant when *p* < 0.05. All statistical analyses were carried out using STATISTICA v.10 StatSoft Inc. 1984–2011 (San Francisco, CA, USA) and GraphPad (San Diego, CA, USA).

## Results

### Effects of repeated administration of 5-MeO-DIPT during adolescence on the basal extracellular levels of DA, 5-HT and glutamate measured in the adulthood (PND 90)

The basal extracellular levels of DA in the rat striatum and nucleus accumbens on PND 90 were significantly (*p* < 0.01) decreased after repeated-intermittent 5-MeO-DIPT administration during adolescence. Similarly, the basal extracellular 5-HT levels were significantly (*p* < 0.01) decreased in the rat striatum and nucleus accumbens. In contrast, the basal extracellular level of glutamate was significantly (*p* < 0.01) increased in the rat nucleus accumbens and frontal cortex (Table 1).

**Table 1:** Basal levels of dopamine (DA), serotonin (5-HT) and glutamate (GLU) in the rat striatum, nucleus accumbens and frontal cortex after repeated administration of 5-methoxy-*N*,*N*-diisopropyltryptamine (5-MeO-DIPT; 8 × 2.5 mg/kg) during adolescence, and measured on PND 90

Experimental group	DA (pg/10 μL)	5-HT (pg/10 μL)	GLU (ng/10 μL)
	Mean ± SEM (*n*)		
Striatum			
Saline	5.32 ± 0.26 (16)	0.94 ± 0.09 (16)	1.58 ± 0.39 (16)
5-MeO-DIPT in adolescence	3.26 ± 0.44 (24)*	0.58 ± 0.04 (24)*	1.49 ± 0.19 (24)
Nucleus accumbens			
Saline	2.15 ± 0.6 (16)	0.57 ± 0.8 (16)	1.36 ± 0.27 (16)
5-MeO-DIPT in adolescence	0.66 ± 0.09 (24)*	0.44 ± 0.03 (24)*	3.22 ± 0.28 (24)*
Frontal cortex			
Saline	0.93 ± 0.16 (16)	0.48 ± 0.05 (16)	1.04 ± 0.24 (16)
5-MeO-DIPT in adolescence	0.96 ± 0.11 (24)	0.46 ± 0.40 (24)	2.98 ± 0.9 (24)*

Data are the mean ± standard error of the mean (SEM). *p < 0.01 vs. respective control (one-way ANOVA and Tukey’s post hoc test)

### Effects of repeated administration of 5-MeO-DIPT during adolescence on extracellular levels of DA, 5-HT and glutamate measured in the adulthood (PND 90) in the rat striatum, nucleus accumbens and frontal cortex

#### Striatum

**Fig. 2:**
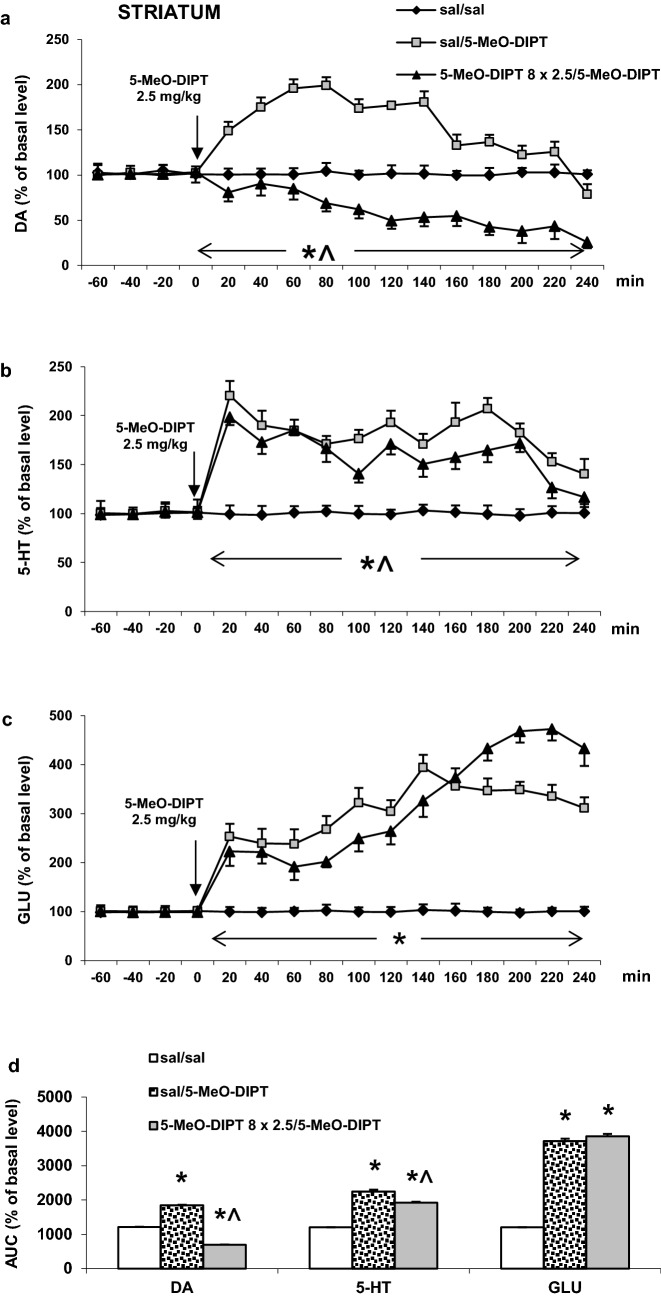
Effects of a challenge dose of 5-MeO-DIPT (2.5 mg/kg) on extracellular levels of dopamine (DA), serotonin (5-HT) and glutamate (GLU) measured in adulthood (PND 90) in the striatum (STR) in rats treated with 5-MeO-DIPT (8 × 2.5 mg/kg) during adolescence. **a**–**c** show each time-course, while **d** shows the total effects expressed as the area under the curve (AUC) of the percent of each basal level. Values are the mean ± standard error of the mean (SEM) [*n* = 4 animals for saline/saline and 6 animals per experimental group (saline/5-MeO-DIPT and 5-MeO-DIPT 8 × 2.5/5-MeO-DIPT)]. The time of drug injection is indicated with an arrow. *p < 0.001 vs. sal/sal group; ^*p* < 0.001 vs. sal/5-MeO-DIPT group (time-course: repeated measures ANOVA and Tukey’s post hoc test; total effect: one-way ANOVA and Tuckey’s post hoc test)

5-MeO-DIPT given repeatedly (2.5 mg/kg/day) for 8 days during adolescence significantly decreased the extracellular DA level in the rat striatum as measured on PND 90 in response to the challenge dose of 2.5 mg/kg (Fig. 2a). The same 5-MeO-DIPT dose markedly increased the extracellular DA level in saline-treated animals (Fig. 2a). Repeated measures ANOVA showed significant effects of treatment groups (*F*_2,11_ = 2677, *p* < 0.0001), sampling period (*F*_11,121_ = 82, *p* < 0.0001) and the interaction between treatment groups and sampling period (*F*_22,121_ = 34, *p* < 0.0002).

The extracellular 5-HT levels in the rat striatum were increased by the challenge 5-MeO-DIPT dose of 2.5 mg/kg both in saline- and 5-MeO-DIPT-treated groups during adolescence; however, response to 5-MeO-DIPT was stronger in saline-treated animals (Fig. 2b). Repeated measures ANOVA showed significant effects of treatment groups (*F*_2,12_ = 488, *p* < 0.0001), sampling period (*F*_11,132_ = 32, *p* < 0.0001) and the interaction between treatment groups and sampling period (*F*_22,132_ = 11, *p* < 0.0001).

The extracellular glutamate levels were increased to a similar extent by the challenge 5-MeO-DIPT dose at 2.5 mg/kg both in saline- and 5-MeO-DIPT-treated groups during adolescence (Fig. 2c). Repeated measures ANOVA showed significant effects of treatment groups (*F*_2,10_ = 915, *p* < 0.0001), sampling period (*F*_11,110_ = 110, *p* < 0.0001) and the interaction between treatment groups and sampling period (*F*_22,110_ = 180, *p* < 0.0001).

The total effects expressed as areas under the curve (AUCs) shown in Fig. 2d reflect the responses to 5-MeO-DIPT resulting in striatal DA, 5-HT and glutamate release presented as time-course curves.

#### Nucleus accumbens

**Fig. 3:**
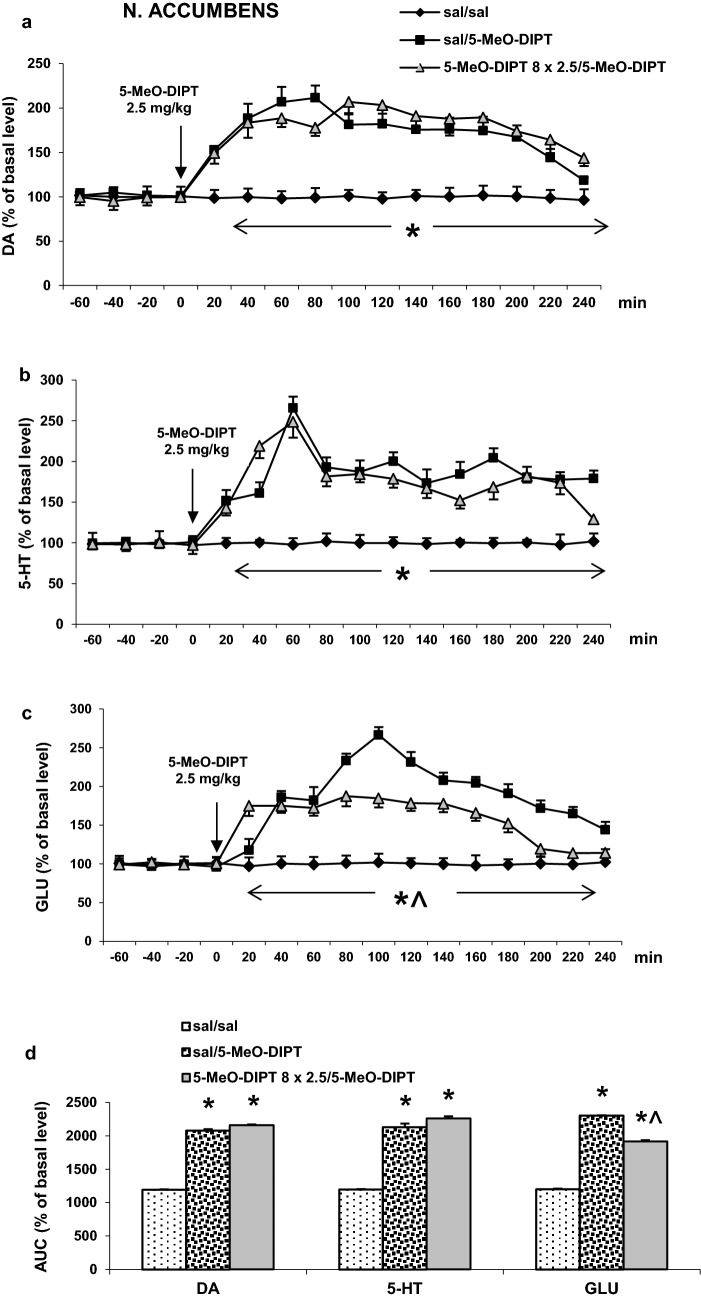
Effects of a challenge dose of 5-MeO-DIPT (2.5 mg/kg) on extracellular levels of DA, 5-HT and GLU measured in adulthood (PND 90) in the nucleus accumbens (NAS) in rats treated with 5-MeO-DIPT (8 × 2.5 mg/kg) during adolescence. **a**–**c** show each time-course, while **d** shows the total effects expressed as the AUC of the percent of each basal level. Values are the mean ± SEM [*n* = 4 animals for saline/saline and 6 animals per experimental group (saline/5-MeO-DIPT and 5-MeO-DIPT 8 × 2.5/5-MeO-DIPT)]. The time of drug injection is indicated with an arrow. *p < 0.001 vs. sal/sal group; ^*p* < 0.001 vs. sal/5-MeO-DIPT group (time-course: repeated measures ANOVA and Tukey’s post hoc test; total effect: one-way ANOVA and Tukey’s post hoc test)

The responses to the challenge dose of 5-MeO-DIPT (2.5 mg/kg) of the extracellular DA level were similar both in saline- and 5-MeO-DIPT-treated animals during adolescence (Fig. 3a). Repeated measures ANOVA showed significant effects of treatment groups (*F*_2,11_ = 1765, *p* < 0.0001), sampling period (*F*_11,121_ = 46, *p* < 0.0001) and the interaction between treatment groups and sampling period (*F*_22,121_ = 18, *p* < 0.0001).

The challenge dose of 5-MeO-DIPT (2.5 mg/kg) increased the extracellular 5-HT levels in the rat nucleus accumbens similarly both in saline- and 5-MeO-DIPT-treated animals during adolescence (Fig. 3b). Repeated measures ANOVA showed significant effects of treatment groups (*F*_2,11_ = 303, *p* < 0.0001), sampling period (*F*_11,121_ = 43, *p* < 0.0001) and the interaction between treatment groups and sampling period (*F*_22,121_ = 18, *p* < 0.0001).

The extracellular glutamate levels in the nucleus accumbens were increased by the challenge dose of 5-MeO-DIPT (2.5 mg/kg) more potently in saline- than in 5-MeO-DIPT-treated animals during adolescence (Fig. 3c). Repeated measures ANOVA showed significant effects of treatment groups (*F*_2,10_ = 5330, *p* < 0.0001), sampling period (*F*_11,110_ = 128, *p* < 0.0001) and the interaction between treatment groups and sampling period (*F*_22,110_ = 97, *p* < 0.0001).

Total effects expressed as AUCs shown in Fig. 3d reflect the responses to 5-MeO-DIPT in accumbal DA, 5-HT and glutamate release presented as time-course curves.

#### Frontal cortex

**Fig. 4:**
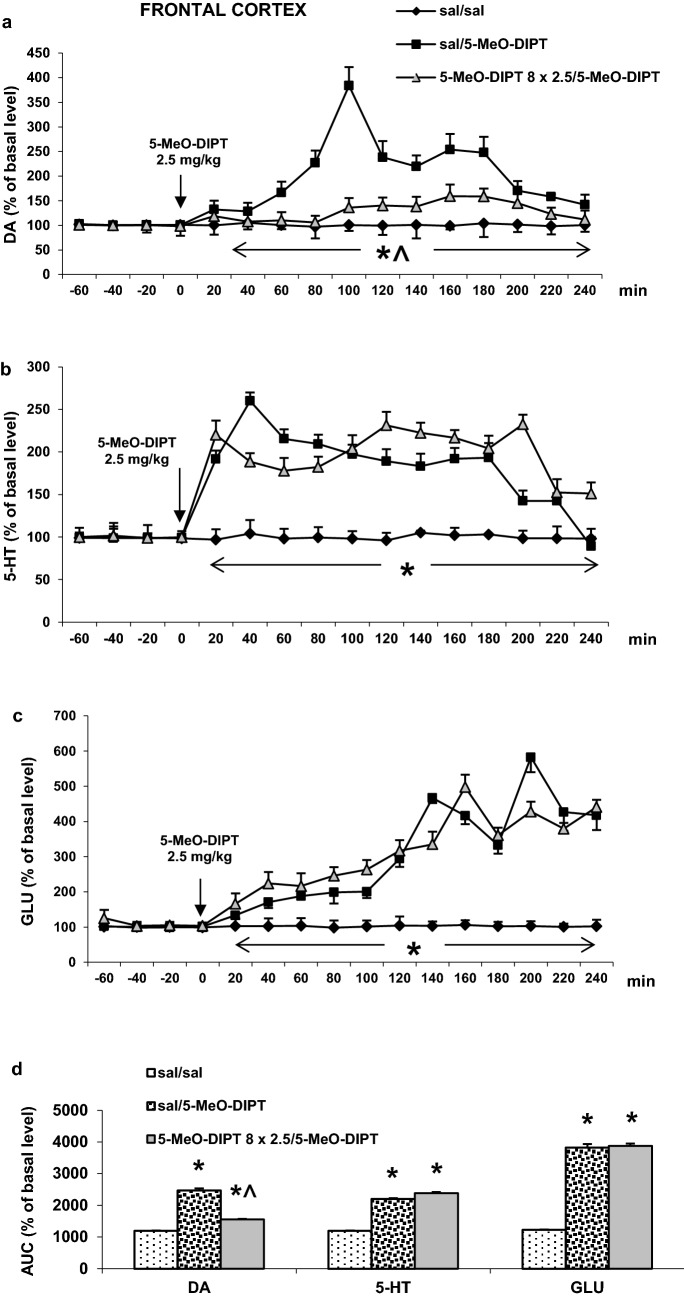
Effects of a challenge dose of 5-MeO-DIPT (2.5 mg/kg) on extracellular levels of DA, 5-HT and GLU measured in adulthood (PND 90) in the frontal cortex (FCX) in rats treated with 5-MeO-DIPT (8 × 2.5 mg/kg) during adolescence. **a**–**c** show the time-courses, while **d** shows the total effects expressed as the AUC of the percent of each basal level. Values are the mean ± SEM [*n* = 4 animals for saline/saline and 6 animals per experimental group (saline/5-MeO-DIPT and 5-MeO-DIPT 8 × 2.5/5-MeO-DIPT)]. The time of drug injection is indicated with an arrow. *p < 0.001 vs. sal/sal group; ^*p* < 0.001 vs. sal/5-MeO-DIPT group (time-course: repeated measures ANOVA and Tukey’s post hoc test; total effect: one-way ANOVA and Tukey’s post hoc test)

The challenge dose of 5-MeO-DIPT (2.5 mg/kg) increased extracellular DA levels in the rat frontal cortex more potently in animals treated with saline only than in those treated with 5-MeO-DIPT during adolescence (Fig. 4a). Repeated measures ANOVA showed significant effects of treatment groups (*F*_2,12_ = 405, *p* < 0.0001), sampling period (*F*_11,132_ = 84, *p* < 0.0001) and the interaction between treatment groups and sampling period (*F*_22,132_ = 59, *p* < 0.0001).

The increase in extracellular 5-HT level in the rat frontal cortex induced by the challenge dose of 5-MeO-DIPT (2.5 mg/kg) was similar in saline- and 5-MeO-DIPT-treated animals during adolescence (Fig. 4b). Repeated measures ANOVA showed significant effects of treatment groups (*F*_2,12_ = 446, *p* < 0.0001), sampling period (*F*_11,132_ = 31, *p* < 0.0001) and the interaction between treatment groups and sampling period (*F*_22,132_ = 24, *p* < 0.0001).

The extracellular glutamate levels were increased by the challenge dose of 5-MeO-DIPT (2.5 mg/kg) to a similar extent in saline- and 5-MeO-DIPT-treated animals during adolescence (Fig. 4c). Repeated measures ANOVA showed significant effects of treatment groups (*F*_2,11_ = 318, *p* < 0.0001), sampling period (*F*_11,121_ = 88, *p* < 0.0001) and the interaction between treatment groups and sampling period (*F*_22,121_ = 27, *p* < 0.0001).

The total effects expressed as AUC shown in Fig. 4d reflect the responses to 5-MeO-DIPT resulting in cortical DA, 5-HT and glutamate release presented as time-course curves.

### Effects of single and repeated administration of 5-MeO-DIPT during adolescence on oxidative DNA damage in the rat cortex

**Fig. 5:**
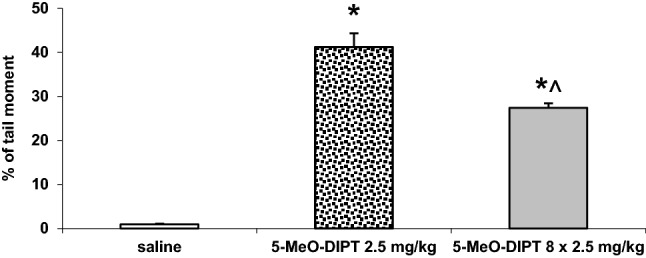
Effects of a single (2.5 mg/kg) and repeated (8 × 2.5 mg/kg) administration of 5-MeO-DIPT during adolescence on the oxidative damage of DNA in nuclei from the rat cortex. Data are the mean ± SEM (*n* = 6 animals per group) and represent an Olive tail moment. Loss of DNA integrity persisted until 60 days after drug administration. *p < 0.01 in comparison to control group, ^*p *< 0.01 repeated vs. single administration (one-way ANOVA and Tukey’s post hoc test)

5-MeO-DIPT given repeatedly (2.5 mg/kg/day) for 8 days during adolescence produced DNA damage shown as a percent of the Olive tail moment in the rat cortex on PND 90 (Fig. 5). However, the damage was smaller in animals repeatedly treated with 5-MeO-DIPT than in animals which received only a single dose (2.5 mg/kg) of 5-MeO-DIPT.

### Effects of repeated administration of 5-MeO-DIPT during adolescence on caspase-3 activities in the striatum, nucleus accumbens and frontal cortex of adult rats

**Fig. 6:**
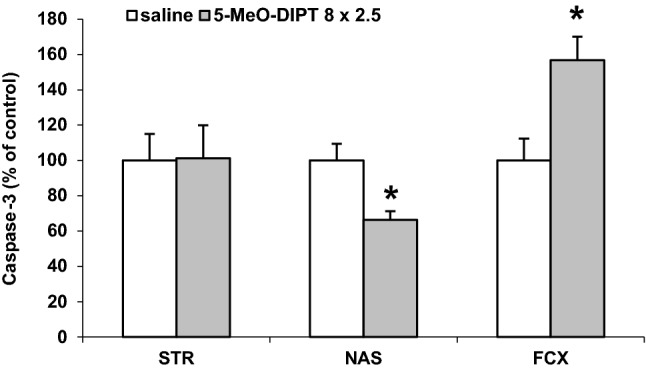
Effects of repeated 5-MeO-DIPT (8 × 2.5 mg/kg) administration during adolescence on caspase-3 activity measured in the rat STR, NAS and FCX in adulthood (PND 90). The results are the mean ± SEM (*n* = 6 animals per group) and are presented as a percent of the saline treated group. *p < 0.01 in comparison to the saline group (one-way ANOVA preceded by the Leven’s test of homogeneity of variances)

Repeated 5-MeO-DIPT administrations during adolescence significantly decreased caspase-3 activity in the nucleus accumbens to 66% of the control level (*p* < 0.01), increased its level to 156% of the control level in the frontal cortex (*p* < 0.01), but had no effect on caspase-3 activity in the striatum as measured on PND 90 (Fig. 6).

### Effects of repeated administration of 5-MeO-DIPT during adolescence on the mRNA expression levels for *Htr1a* and *Htr2a* genes, and *Gpx3* and *Sod1* genes in the rat frontal cortex and striatum, respectively

**Fig. 7:**
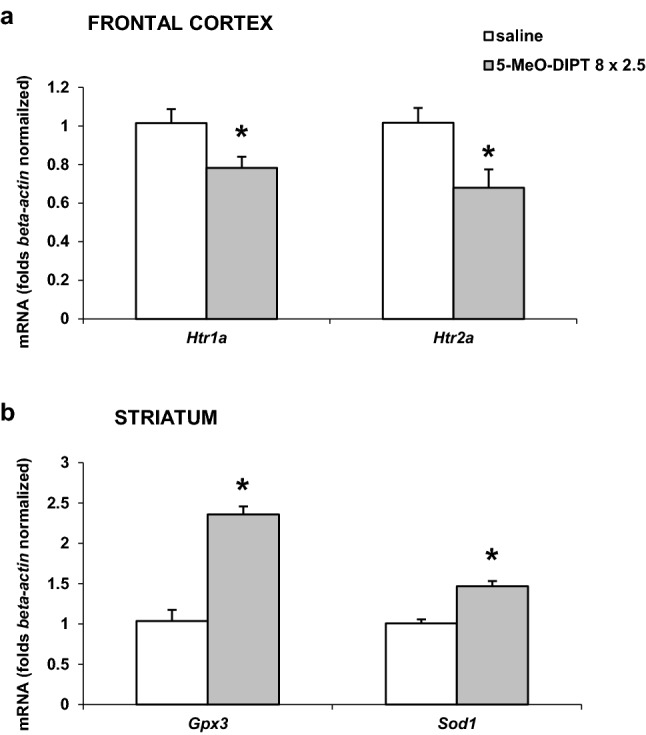
Effects of repeated 5-MeO-DIPT (8 × 2.5 mg/kg) administration during adolescence on the mRNA expression levels of *Htr1a* and *Htr2a* genes (**a**) and *Gpx3* and *Sod1* genes (**b**) measured in adulthood (PND 90) in the rat FCX and STR, respectively. The open bars show the saline control, and the gray bars the results with the repeated administration of 5-MeO-DIPT (8 × 2.5 mg/kg) during adolescence. Each bar represents the mean ± SEM of three independent experiments from six rats. The number of replicates for each experiment ranged from two to three. *p < 0.05 vs. saline group (one-way ANOVA preceded by the Levene’s test of homogeneity of variances)

Repeated 5-MeO-DIPT administration during adolescence significantly (*p* < 0.05) decreased mRNA expression levels of *Htr1a* and *Htr2a* genes in comparison to the respective controls when measured on PND 90 in the rat frontal cortex (Fig. 7a). Data were normalized to those of *β*-*actin.*

Repeated 5-MeO-DIPT administration during adolescence significantly (*p* < 0.05) increased mRNA expression levels of *Gpx3* and *Sod1* genes in comparison to the respective controls as measured on PND 90 in the rat striatum (Fig. 7b). Data were normalized to those of *β*-*actin.*

### Effects of 5-MeO-DIPT and methamphetamine on viability of neuroblastoma SH-SY5Y and Hep G2 cells

**Fig. 8:**
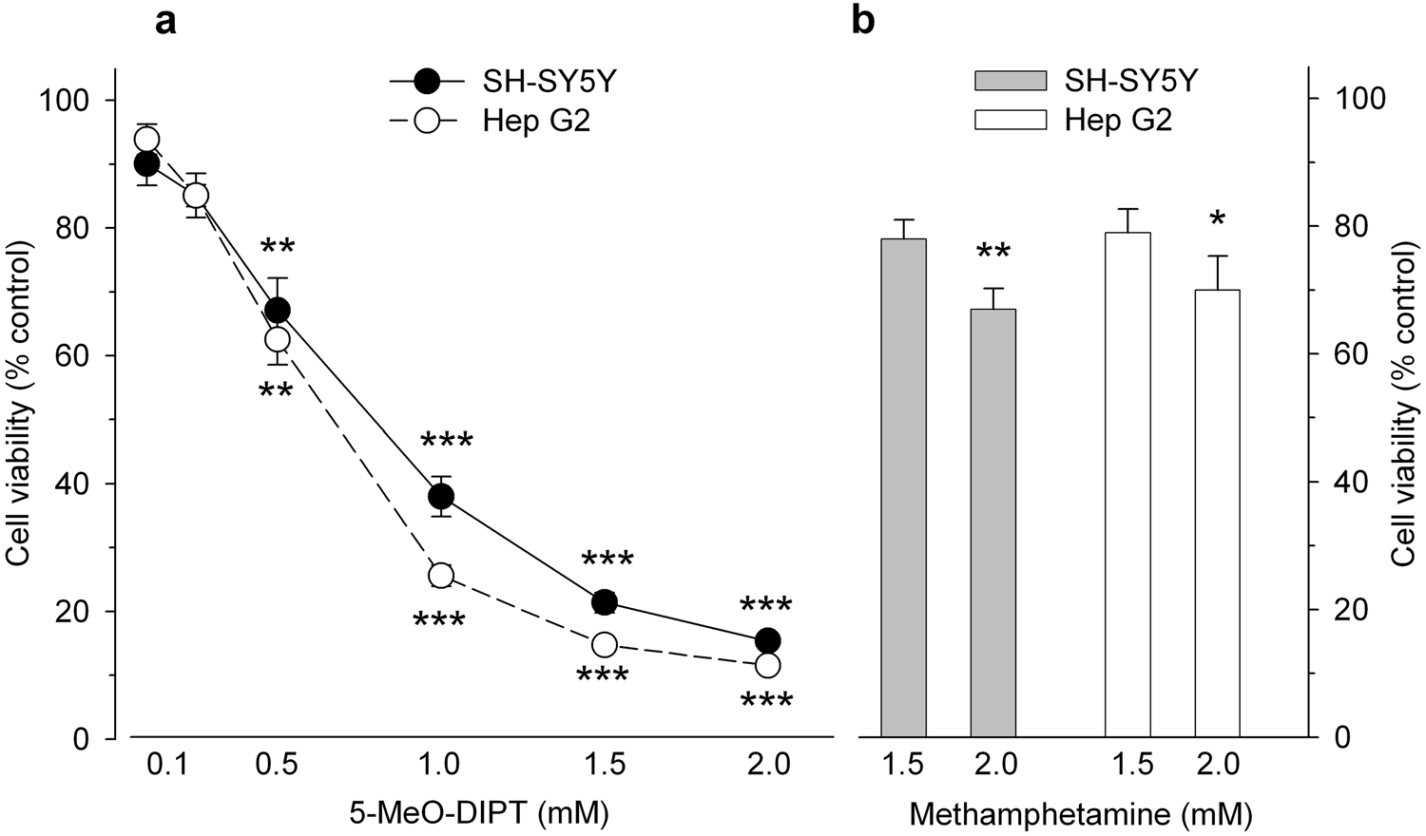
Effects of 5-MeO-DIPT (**a**) and methamphetamine (**b**) on viability of neuroblastoma SH-SY5Y and Hep G2 cells. The cells were incubated with the tested compounds for 24 h and cell viability and mitochondrial function was analyzed by the MTT test. Data are the mean ± SEM of 10–12 values per group and are expressed as a percentage of the respective control. *p < 0.05, **p < 0.01, ***p < 0.001 vs. control (one-way ANOVA followed by Dunnett’s post hoc test)

Cultured SH-SY5Y and Hep G2 cell lines were challenged with 5-MeO-DIPT (0.1–2 mM) for 24 h and cell viability was measured by mitochondrial activities. Exposure of both cell lines to 5-MeO-DIPT resulted in a concentration-dependent decrease of their viability with statistical significance starting at 0.5 mM for both cell lines. The maximal effect was observed at 2 mM, i.e., decrease in survival of SH-SY5Y and Hep G2 cells to 15 and 11% of control values, respectively (Fig. 8a). The calculated EC_50_ values were 0.8 mM for SH-SY5Y cells and 0.6 mM for Hep G2 cells.

Methamphetamine, used as a reference compound, induced a mark decrease in cell viability. However, the cytotoxic effect of methamphetamine was much less pronounced than that of 5-MeO-DIPT and statistical significance was reached only at 2.0 mM, where 24-h exposure led to a decrease of cell survival to 70 and 67% of control values, for Hep G2 and SH-SY5Y cells, respectively (Fig. 8b).

## Discussion

5-MeO-DIPT given repeatedly to animals during adolescence changed response of adult animals to the challenge dose of the drug. It decreased response of DA neurons in the striatum (Fig. 2a) and frontal cortex (Fig. 4a). The response of 5-HT and glutamatergic neurons was also decreased in the striatum (Fig. 2b, c) and nucleus accumbens (Fig. 3b, c), respectively. The basal extracellular levels of DA and 5-HT were decreased in the striatum and nucleus accumbens after repeated 5-MeO-DIPT administration during adolescence. In contrast, the basal extracellular glutamate level was markedly increased in the nucleus accumbens and frontal cortex (Table 1). The damage of cortical DNA (Fig. 5), increased expression of antioxidant enzymes (Fig. 7b), and changes in caspase-3 activity (Fig. 6) were also observed in adult animals after repeated 5-MeO-DIPT administration during adolescence. Furthermore, decreased expression of 5-HT_1A_ and 5-HT_2A_ receptors was observed (Fig. 7a). Marked cytotoxicity of 5-MeO-DIPT in the SH-SY5Y and Hep G2 cell line was found (Fig. 8a).

In our study, we observed changes in basal extracellular levels of neurotransmitters which persisted until adulthood in animals pretreated with 5-MeO-DIPT. These changes were not identical for all neurotransmitters and all studied brain regions. Namely, basal DA level was decreased in the striatum and nucleus accumbens; similarly basal 5-HT level was also decreased in the same brain regions. On the other hand, basal glutamate level was increased in the frontal cortex and nucleus accumbens (Table 1). It may be hypothesized that repeated-intermittent administration of 5-MeO-DIPT caused deficit in DA and 5-HT nerve endings in subcortical brain regions. In contrast, we observed activation of excitatory neurons in the frontal cortex and nucleus accumbens resulting in outflow of glutamate from glutamatergic neuronal cells or astrocytes by repeated-intermittent administration of 5-MeO-DIPT. The overstimulation of cortical glutamatergic cells could underlie the excitotoxicity leading to oxidative damage of neuronal cells shown by single- and double-strand DNA breaks and activation of caspase-3 activity. It indicates development of oxidative stress which elicits impairment of glutamatergic neurons in the cortex. As a consequence of glutamatergic cell impairment, stimulatory input to the ventral tegmental area (VTA), substantia nigra and nuclei raphe could be weaker [28, 29], which resulted in decreased basal activities of DA and 5-HT neurons in subcortical regions.

Another issue is the altered neuronal response to the challenge dose of 5-MeO-DIPT, e.g., weaker responses of DA neurons in the striatum (Fig. 2a) and frontal cortex (Fig. 4a), 5-HT neurons in striatum (Fig. 2b) and glutamatergic neurons in the nucleus accumbens (Fig. 3c). These results may be explained by the decreased expression of cortical 5-HT_1A_ and 5-HT_2A_ receptors observed in our study. 5-HT_2A_ receptors are located on cortical pyramidal cells as well as on the DA cell bodies in the VTA, where they regulate activities of pyramidal cells and mesolimbic and mesocortical DA pathways, respectively [28, 30–33]. 5-HT_1A_ receptors, besides the somatodendritic location in the dorsal raphe nucleus, are also found postsynaptically in the limbic and cortical regions [34, 35]. The decreased expression of 5-HT_2A_ receptors in pyramidal cells of adult animals shown in our study after repeated treatment with 5-MeO-DIPT during adolescence may be the cause of weaker response of 5-HT and DA neurons to the challenge dose of this drug. Similarly, we observed the decreased expression of cortical 5-HT_1A_ receptors in adult animals after repeated treatment with 5-MeO-DIPT during adolescence (Fig. 7a). 5-HT_1A_ receptor stimulation leads to inhibition of neuronal activity [34]. These receptors were shown to be located on GABAergic interneurons in the frontal cortex [36]. Thus, the possibility of decreased density as shown by attenuated expression of inhibitory 5-HT_1A_ receptors and subsequent increase in GABA levels may result in weaker responses of DA neurons in the striatum (Fig. 2a) and frontal cortex (Fig. 4a) and 5-HT neurons in the striatum (Fig. 2b) of adult animals to the challenge dose of 5-MeO-DIPT.

The attenuated response of glutamatergic neurons in the nucleus accumbens (Fig. 3c) may be related to damage of the cortico-accumbal glutamatergic pathway or weaker direct stimulation of these efferents by 5-MeO-DIPT via 5-HT_2A_ receptors, the cortical expression of which was decreased (Fig. 7a). Interestingly, the damage of nuclear DNA in animals treated repeatedly with 5-MeO-DIPT in adolescence was less pronounced than in animals which received a single dose of the drug (Fig. 5). This effect suggests a possible activation of the enzymatic defense systems during repeated administration of 5-MeO-DIPT. In the striatum of adult animals, the caspase-3 activity was unchanged (Fig. 6), but expression of *Gpx3* and *Sod1* was increased (Fig. 7b). It is likely that the enzymatic defense system efficiently protected striatal cells from apoptotic cell death in response to 5-MeO-DIPT given during adolescence [37]. The response of cells to oxidative stress may involve post-translational modifications of many proteins and their functions. Caspase-3 may be, for instance, regulated by glutathionylation resulting from the oxidation of the major cellular antioxidant glutathione (GSH). The oxidized form of GSH, glutathione disulfide, was shown to inhibit caspase-3 activity in HL-60 cells [38]. This mechanism may underlie the inhibition of caspase-3 in the nucleus accumbens by 5-MeO-DIPT. The issue of defense system activation by 5-MeO-DIPT needs further investigation.

Cytotoxic properties of 5-MeO-DIPT were shown by its effect on cell viability in SH-SY5Y and Hep G2 cell lines. We found that in the presence of 2 mM concentration of 5-MeO-DIPT, only ca. 15% of cells survived, while in the same concentration of methamphetamine, more than 70% of cells survived in both cell lines (Fig. 8). These data indicate a potent direct cytotoxicity of 5-MeO-DIPT, even stronger than cytotoxicity produced by methamphetamine. The mechanism of a potent cytotoxic effect of 5-MeO-DIPT is not clear and needs further studies.

Adolescence is a very important period for brain development and there are strong evidences demonstrating that abnormalities during neurodevelopment, including exposure to drugs of abuse, may lead to neuropsychiatric disorders. Case reports showed marked 5-MeO-DIPT toxicity and indicated long-lasting changes in brain function reflected by hallucinogen-persisting perception disorder development or prolonged delusional state [39, 40]. Our study confirms the neurotoxic properties of 5-MeO-DIPT found in animal experimental models.

## Conclusions

5-MeO-DIPT exposure during adolescence resulted in a marked impact of this substance on DA, 5-HT and glutamate neurotransmission. 5-MeO-DIPT induced oxidative stress which was manifested by oxidative damage of nuclear DNA, activation of apoptotic signals and increased expression of antioxidant enzymes. Moreover, a dramatic in vitro cytotoxicity of 5-MeO-DIPT was observed.
